# THE IMPLEMENTATION OF GERIATRIC EMERGENCY DEPARTMENT ACCREDITATION (GEDA) IN RURAL CRITICAL ACCESS HOSPITALS

**DOI:** 10.1093/geroni/igac059.1334

**Published:** 2022-12-20

**Authors:** Ellen Flaherty, Kevin Biese

## Abstract

The largely rural setting of Northern New England offers unique challenges to implementing improved acute care for the growing geriatric population. Northern New England is one of the United States’ most rapidly aging regions, with Vermont and New Hampshire being the second and third oldest US states respectively by median age (U.S. Census 2017). There is a need to expand innovations in geriatric emergency medicine to reach older adults in rural areas such as Northern New England. Dartmouth-Hitchcock Medical Center and the West Health are collaborating on a project leveraging telehealth to extend the reach of a GED to rural hospitals, as well as investigate the opportunities for scaling and sustaining this concept to other rural facilities across Northern New England and throughout the country. This symposium will focus on our experience implementing a hub and spoke model to achieve our goal of improving the care of older adults in rural emergency departments.

